# Advances of Endothelial Progenitor Cells in the Development of Depression

**DOI:** 10.3389/fncel.2021.608656

**Published:** 2021-08-05

**Authors:** Nana Yang, Shiyu Sun, Guangqing Duan, Kaixuan Lv, Chen Liang, Linlin Zhang, Jielun Yu, Yaohui Tang, Guohua Lu

**Affiliations:** ^1^School of Bioscience and Technology, Weifang Medical University, Weifang, China; ^2^Medical Laboratory Animal Center, Weifang Medical University, Weifang, China; ^3^School of Clinical Medicine, Weifang Medical University, Weifang, China; ^4^School of Chemical Engineering, Qingdao University of Science & Technology, Qingdao, China; ^5^Med-X Research Institute and School of Biomedical Engineering, Shanghai Jiao Tong University, Shanghai, China; ^6^School of Psychology, Weifang Medical University, Weifang, China

**Keywords:** endothelial progenitor cells, depression, psychological disease, cardiovascular disease, TNF-α

## Abstract

Depression is a major psychological disease of human beings. With the severity of depression, it elevates the risk of cardiovascular disease (CVD), especially acute coronary syndrome (ACS), resulting in serious harm to human health. The number of endothelial progenitor cells (EPCs) is closely related to the development of depression. It has been reported that the number of peripheral blood EPCs in patients with depression was reduced. However, effects on the function of EPCs in depression are still unclear. This paper aims to analyze and summarize the research of EPCs in depression, and we envision that EPCs might act as a new target for evaluating the severity of depression and its complications.

## Introduction

Depression is one of the most common psychological disorders which acts as the primary risk factor for suicide and cardiovascular disease (Shi et al., 2017). As a common disease detrimental to the physical and mental health of human beings, depression is prominently manifested as depressive symptoms, such as reduced activity, memory loss, fatigue, sleep disorders, self-abandonment and even suicide (Malhi and Mann, 2018). Data shows that people aged 55–64 have the highest incidence of depression. In addition, depression is associated with educational level and unhealthy living habits which also serve as an important cause of CVD (Anda et al., 1993; Klakk et al., 2018). Therefore, effective means of evaluating or treating depression have become the main goal of depression research.

EPCs, also known as a precursor of vascular endothelial cells, have the ability to proliferate, migrate, differentiate and form new blood vessels *in vivo* (Kaushik and Das, 2019). Under the stimulation of physiological or pathological factors, EPCs can be migrated from bone marrow to peripheral blood and participate in the repair of damaged blood vessels. Moreover, the proliferation and differentiation of EPCs are vital to tumor angiogenesis. Recently scientists proposed a certain correlation between EPCs and depression (Dome et al., 2009; Di Stefano et al., 2014; Felice et al., 2015). It becomes the most eye-catching whether EPCs can serve as an indicator for the severity of depression.

## Characteristics of EPCs

Asahara et al. (1997) discovered precursor cells capable of differentiating into vascular endothelial cells in circulating peripheral blood and named them as vascular EPCs for the first time in 1997. Since then, the characteristics, biological functions and potential therapeutic application of EPC have become hot topics in the field. Functional assays and surface marker-based molecular definition are two essential ways to characterize EPCs. Numerous assays were used to evaluate self-renewal capacity and potency of EPCs, which are crucial to functionally define and classify EPCs (Patel et al., 2016). In addition, it was found that these cells during *in vitro* culture could co-express vascular endothelial growth factor receptor2 (VEGFR-2) and a large number of cell surface markers including CD34, CD133 and von Willebrand factor (VWF), and have the potential to differentiate into mature endothelial cells. Afterward, CD133^+^/CD34^+^/VEGFR2^+^/CD45^–^ was usually used as the surface marker of EPCs (Pulito-Cueto et al., 2020). The discovery of cell surface markers played an important role in the investigation of human cardiovascular and cerebrovascular diseases and malignant tumors (Chong et al., 2016).

EPCs are usually classified into two types named early EPCs (eEPCs) and late EPCs (lEPCs) (Kou et al., 2020). eEPCs appeared 3–5 days after the onset of *in vitro* colony-forming assays from peripheral blood cells while 2–3 weeks needed for lEPCs (Yuan et al., 2017). Besides the difference on culturing time, cell origin, surface markers, biological functions and further applications are quite distinctive between these two subpopulations. In brief, eEPCs express typical hematopoietic marker CD133 and CD45, monocyte marker CD14 and also endothelial marker like VEGFR2, but negative for CD34, which are considered to have low proliferation and colony-forming abilities but could release numerous cytokines (Ormiston et al., 2015). In contrast, lEPCs are lack of hematopoietic markers CD133 and CD45, but positive for CD34 and VEGFR2 (Yoon et al., 2005). With the high proliferative potential and colony-forming ability, lEPCs could promote vessel formation through angiogenetic pathway (Prasain et al., 2012). EPCs are heterogeneous populations *in vivo*. Thus, it is critical to properly classify EPCs while analyzing them in disease models.

EPCs are rare in peripheral blood but relatively abundant in bone marrow in healthy people. Under a pathological state, EPCs can be migrated from bone marrow to peripheral blood, and promote angiogenesis. For example, in the case of tumorigenesis, tumor tissues can secrete VEGF, SDF-1, G-CSF, GM-CSF and other cytokines and chemokines which mobilize EPCs from bone marrow to tumor site, and participate in tumor angiogenesis (Rana et al., 2018). The aforementioned recruitment of EPCs can also be promoted by some stimulants, such as fibroblast growth factor, epidermal growth factor, and estrogen (Capillo et al., 2003; Vega et al., 2017). Some diseases can be treated or alleviated by changing the number and function of EPCs. For example, EPCs can be employed to repair the heart of patients with myocardial infarction (Bianconi et al., 2018) and improve lung function (Li et al., 2013; Salter and Sehmi, 2017) as well as the angiogenic ability of patients with diabetes (Altabas, 2015). Tagawa et al. (2015) and Morishita et al. (2016) found the number EPCs could be used as an important index to predict the incidence of CVD. EPCs possess a broad application prospect in investigating angiogenesis and cellular treatment of CVD due to their autologous isolation, amplification and transplantation, which is free of rejection reaction.

## Effect of Depression on the Quantity of EPCs

The quantity of EPCs is an important index to predict the occurrence and development of various diseases, so what changes will happen to EPCs in patients with depression. Can the quantitative changes in EPCs be a predictor for depression. And how depression affects the quantity of EPCs. Studies of the effect of depression on the number of EPCs are shown in Table 1. A total of 10 articles on endothelial progenitor cells and depression were searched in national center for biotechnology information (NCBI). Four papers are associated with depression and the number of EPCs, three articles are related with EPCs numbers after treatment with drugs of depression, and three papers show the correlation between depression, cardiovascular disease and the number of EPCs.

**TABLE 1:** Studies of the effect of depression on the number of EPCs.

EPCs markers	Methods of calculating the number of EPCs	Key fingdings	References
CD34^+^/VEGFR2^+^ CD133^+^/VEGFR2^+^	The number of cells per milliliter of blood	The number of mature (CD34^+^ /VEGFR2^+^) and immature (CD133^+^/VEGFR2^+^) EPCs were significantly decreased in depression patients, and EPCs levels was significant inverse relationship with the severity of depressive symptoms.	Dome et al., 2009
CD34^+^/VEGFR2^+^ CD133^+^ /VEGFR2^+^	The number of EPCs was expressed as absolute EPCs counts divided by the lymphocyte counts.	The percentage of circulating CD34^+^/KDR^+^ EPCs was lower in high depression score than in normal depression score. And the percentage of circulating CD133^+^/KDR^+^ EPCs was no different between two groups.	Chen H. et al., 2011
CD34^+^/KDR^+^/CD133^+^	Absolute number of cells per ml of blood	Levels of circulating CD34^+^CD133^+^KDR^+^ EPCs and endothelial colony-forming units in patients with depression were lower than that of healthy subjects.	Yang et al., 2011
CD34^+^/VEGFR2^+^	The number of EPCs was quantified as the number of these cells per 10^6^ lymphocyte.	There was no significant alteration in CEPCs levels in the course of recovery from major depression.	Dome et al., 2012
CD34^+^/VEGFR2^+^ CD133^+^/VEGFR2^+^	The number of EPCs was expressed as absolute EPCs counts divided by the lymphocyte counts.	In stable angina patients, percentage of circulating CD34^+^/VEGFR2^+^ EPCs and artery flow-mediated dilation in Subjects with high depression or stress score were significantly lower than that in subjects with normal depression or stress score.	Chen et al., 2013
CD34^+^/KDR^+^/CD133^+^	Absolute number of cells per ml of blood	The number of EPCs in acute coronary syndrome with major depressive episode showed significant decrease compared with that in acute coronary syndrome without major depressive episode.	Di Stefano et al., 2014
CD34^+^/KDR^+^/CD133^+^	Absolute number of cells per ml of blood	Circulating CD34^+^CD133^+^KDR^+^ EPCs levels in acute coronary syndromes with affective disorders was significantly lower than that in acute coronary syndromes without affective disorder.	Felice et al., 2015
CD34^+^/CD133^+^	Absolute number of cells per ml of blood	EPCs significantly decreased with the year of intership stress, while the development of depressive symptoms had no significant relationship with changes in EPCs.	Fiedorowicz et al., 2015
CD45^–^/CD146 ^+^/CD31^+^ CD45^–^CD34^+^/KDR^+^	Absolute number of cells per ml of blood	CECs (CD45^–^/CD146^+^/CD31^+^) counts, soluble VWF and VCAM-1 were statistically increased in diagnosis (MD-0) and gradually decreased during treatment. Conversely, EPCs (CD45^–^/CD34^+^/KDR^+^) levels were lower in MD-0, tending to increase throughout treatment.	Lopez-Vilchez et al., 2016
CD133^+^/VEGFR2^+^	the mononuclear cells (lymphocytes, monocytes, and blasts) were set to gates, then the percentage of CD133^+^/ VEGFR2^+^ cells in the mononuclear cells was acquired.	CEPCs levels in blood had no significant difference in chronic mild stress (CMS) group, high-fat diet group, high-fat diet with CMS group, and the group of imipramine and pentoxifylline treatment. Chronic pentoxifylline treatment was more effective in increase CD133^+^ and VEGFR2^+^ cells in rat thoracic aortae.	Labib et al., 2019

Dome et al. (2009) reported significant lower quantities of mature EPCs (CD34^+^/VEGFR2^+^) and immature EPCs (CD133^+^/VEGFR2^+^) in the peripheral blood of patients with depression than those of control by flow cytometry. Since smoking could lead to significantly reduced quantity of circulating EPCs, the smoking habits of patients and control were matched in the study, demonstrating significant lower levels of mature EPCs in smokers than those in non-smokers from both control and patient groups. Although significantly decreased number of EPCs has been observed in patients with depression, the underlying mechanism still needs further elucidation. That may involve in reduced recruitment as well as the capabilities of survival and differentiation of EPCs. Chen H. et al. (2011) not only investigated the changes of EPCs numbers in peripheral blood but also the vascular function and mental state of patients with depression. Their data showed no significant difference in age, gender, hypercholesterolemia prevalence, smoking, systolic blood pressure, diastolic blood pressure, BMI and serum total cholesterol between the patients with higher and normal depression scale scores. Patients from these two groups received similar medications of antidepressants, antihypertensives and statins. Compared with normal depression score (DS), patients with high DS exhibited significantly lower brachial artery flow-mediated dilation (FMD) and the percentage of CD34^+^/KDR^+^ EPCs, with no significant difference in the percentage of circulating CD133^+^/KDR^+^ EPCs (*P* > 0.05). Yang et al. (2011) also investigated that levels of circulating CD34^+^CD133^+^KDR^+^ EPCs and endothelial colony-forming units (CFUs) in patients with depression were lower than that of healthy subjects. EPCs significantly decreased, while the development of depressive symptoms had no significant relationship with changes in EPCs through the year of internship stress (Felice et al., 2015). Altogether, these results indicate that EPCs numbers in peripheral blood were significantly more in healthy subjects than in subjects with depression, and patients with high DS exhibited significantly lower percentage of CD34^+^/KDR^+^ EPCs compared with normal DS, although the methods of calculating EPCs numbers were different in above studies. Large sample of clinical studies on the correlation between depression and EPCs numbers need to be further studied through consistent method of calculating EPCs.

In order to further clarify the changes of EPCs during the treatment of depression, Dome et al. (2012) evaluated the changes of EPCs in peripheral blood during the recovery of the patients. After receiving antidepressant treatment, the severity of patients with depression was significantly improved through the measurement of Montgomery as berg expression rating scale (MADRS), with improved level of cholesterol but no significant changes in the quantity of EPCs. Recent findings showed that CECs (CD45^–^/CD146^+^/CD31^+^) counts, soluble VWF and VCAM-1 were statistically increased in diagnosis (MD-0) and gradually decreased during the selective serotonin reuptake inhibitor escitalopram treatment. Conversely, EPCs (CD45^–^/CD34^+^/KDR^+^) levels were lower in MD-0, tending to increase throughout escitalopram treatment. However, the increase level did not reach statistical significance after 24 weeks of antidepressant treatment (Lopez-Vilchez et al., 2016). Sera from patients with depression damage the endothelial cells *in vitro*, such as increased level of intercellular adhesion molecule-1 (ICAM-1), lower level of endothelial nitric oxide synthase (eNOS) and higher reactive oxygen species (ROS) production. There is no damage to endothelial cells in the serum of the depressed patients with escitalopram treatment for 24 weeks. The methods of calculating EPCs numbers and anti-depression treatments were different in above both studies. CEPCs levels in blood had no significant difference in chronic mild stress (CMS) group, high-fat diet group, high-fat diet with CMS group, and the group of imipramine and pentoxifylline treatment. Chronic pentoxifylline treatment was more effective in increase CD133^+^ and VEGFR2^+^ cells in rat thoracic aortae (Labib et al., 2019).

The percentage of CD34^+^/KDR^+^EPCs (0.029%) in lymphocytes was very low (Vasa et al., 2001), thus the level of EPCs in peripheral blood was difficult to measure and the measurement methods were different. The number of EPCs was quantified as the number of these cells per 10^6^ lymphocytes in Dome et al. (2012) research, while calculating EPCs numbers in Lopez-Vilchez et al. (2016) experiment was used absolute number of cells per ml of blood. This may be the reason why the results of the above two clinical experiments are different. In addition, at least 10^6^ mononuclear cells in peripherals blood were collected in other study, and EPCs numbers were reported as a percentage of mononuclear cells (Wang et al., 2020). Because of low level of EPCs in peripheral, direct count following with ISHAGE gating would be the promising way.

## The Mechanism of Depression Affecting the Quantity and Function of EPCs

### Depression May Affect the Quantity and Function of EPCs by Affecting Levels of Inflammatory Mediators

Depression has sustained a series of inflammatory state, and promoted the increase of inflammatory markers, such as tumor necrosis factor-α (TNF-α), C-reactive protein (CRP) and interleukin 6 (IL-6) (Nukina et al., 2001; Pikhart et al., 2009; Liu et al., 2012). Miller et al. (2005) proposed the participation of tumor necrosis factor-α (TNF-α) in the pathogenesis of depression. TNF-α is a multifunctional cytokine that can directly kill tumor cells and can be up-regulated in the case of depression, leading to functional decline in the peripheral immune system (Liu et al., 2012). Afterward, lots of researchers were performed to investigate whether there was a correlation between depression and TNF-α level. Haapakoski et al. (2015) and Ma et al. (2016) found higher TNF-α levels in the peripheral blood of patients with depression than that of control. Similar results were also reported by Fan et al. (2017) from a study with 64 depression patients and 80 healthy controls. Elevated mRNA and protein levels of TNF-α were found in patients with recurrent depressive disorder (RDD), compared with control (Bobińska et al., 2017). Therefore, it could be concluded that the level of TNF-α in the peripheral blood of patients with depression is higher than that of control.

Several studies have reported that the concentration of CRP and IL-6 was higher in depressed patients than in healthy controls. Depression score was positively related to the levels of CRP in a linear manner. After controlling for confounders, the levels of CRP in subjects with depression were higher than that in healthy controls (Pikhart et al., 2009). Another study showed that the levels of fasting CRP were significantly increased in remitted women with major depressive disorder (MDD) versus controls (Kling et al., 2007). High levels of IL-6 in childhood are associated with an increased risk of depression and psychotic experiences (PEs) in a dose-dependent manner in young adulthood (Khandaker et al., 2014). In depression animal models, studies found that restraint stress stimulated the increased levels of IL-6 (Nukina et al., 2001), and administration of LPS or recombinant IL-6 induced depressive-like behaviors (Dantzer et al., 2008; Hayley et al., 2008; Fu et al., 2010; Sukoff Rizzo et al., 2012). Chourbaji et al. (2006) indicated that *IL-6*-deficient mice were resistant to the stress induced by the development of a depressive-like behaviors.

As a cytokine with many biological effects, TNF-α can reduce the number of EPCs in peripheral blood and reduce the function of EPCs. Chen T. G. et al. (2011) investigated the effects of TNF-α (0, 10, 20, 50, and 100 mg/L, respectively) on the proliferation, migration and adhesion of EPCs isolated from human peripheral blood. The results demonstrated that TNF-α significantly reduced the quantity, capabilities of proliferation, migration and adhesion of EPCs, with a negative correlation between the quantity and function of EPCs and concentration of TNF-α. TNF-α could reduce the number of EPCs in peripheral blood of mice in high fat diet mice, and inhibit the proliferation, migration and angiogenic function of EPCs, which could be alleviated by ApoAI analog peptide of Rev-D-4F (Nana et al., 2015). Likewise, CRP significantly inhibited EPCs migration, adhesiveness and proliferation through receptors for advanced glycation end products (RAGE) (Chen et al., 2012), and induced EPCs apoptosis and necrosis (Fujii et al., 2006). Although IL-6 enhances EPCs migration, proliferation, and differentiation (Fan et al., 2008), bone marrow CD34^+^ cell levels were inversely associated with the inflammatory marker IL-6 in critical limb ischemia patients (Teraa et al., 2013), and the number of EPCs in peripheral blood was negatively correlated with IL-6 levels in rheumatoid arthritis patients (Herbrig et al., 2006).

**FIGURE 1:**
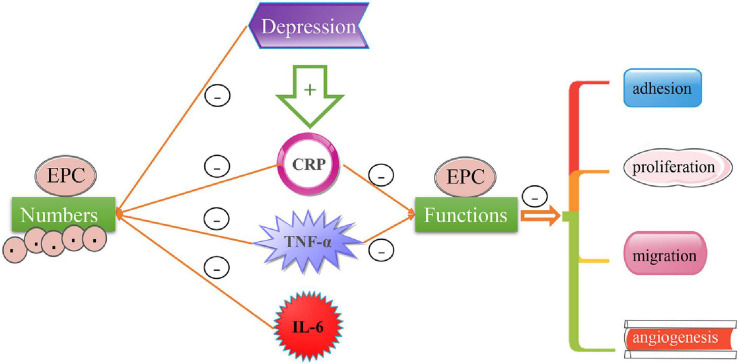
The effect of depression on EPCs numbers and functions. The level of TNF-α, CRP, and IL-6 in peripheral blood of patients with depression are higher than that of healthy subjects, and the level of TNF-α, CRP, and IL-6 have a negative correlation with EPCs numbers. TNF-α and CRP can inhibit the functions of EPCs, such as adhesion, proliferation, migration and angiogenesis *in vitro* study. Whether depression can affect the above functions of EPCs through TNF-α or other inflammatory factors will require further studies.

Despite the decreased number of EPCs and endothelial CFUs in peripheral blood in patients with depression (Yang et al., 2011; Blum et al., 2017), no relevant report on their proliferation, migration and adhesion in patients with depression has been reported to the best of our knowledge. In subjects with depression, the level of TNF-α (Dome et al., 2009; Yang et al., 2011; Labib et al., 2019) and IL-6 (Yang et al., 2011) had a negative correlation with the number of EPC. And there are no relevant studies on whether these inflammatory factors affect endothelial progenitor cell functions in subjects with depression. In consideration of elevated TNF-α in the peripheral blood of patients with depression and its detrimental effects on the proliferation, migration, adhesion of EPCs and angiogenesis, we speculate that depression may affect the function of EPCs by regulating the levels of TNF-α and some inflammatory mediators in the peripheral blood of patients with depression (see Figure 1).

### The Occurrence of Depression Complications May Affect the Quantity and Function of EPCs

Depression can lead to many complications such as CVD and diabetes (Ariyo et al., 2000; Bãdescu et al., 2016), and McClung et al. (2005) and Aragona et al. (2016) found that these complications can affect the quantity and function of EPCs. Therefore, the direct or indirect effects of depression on the quantity and function of EPCs are worth further investigations. Ariyo et al. (2000) proposed that depression increased the probability of patients to suffer from cardiovascular disease and performed a 6 years’ follow up of 4,439 patients with different degrees of depression. It was found that the incidence of coronary heart disease (CAD) was positively correlated with the severity of depression after 6 years, with every 5 scores increase in depression MADRS evaluation corresponding to increased incidence of CAD by 15 % and females exhibiting high incidence than males. In comparison with control, it can be found significantly decreased quantity of EPCs in patients with CAD and the changes in quantity of EPCs serves as an important index to predict the occurrence and development of cardiovascular diseases in the future (McClung et al., 2005; Di Stefano et al., 2014; Felice et al., 2015). Schmidt-Lucke et al. (2019) studied 120 patients consisting of 43 healthy controls, 44 patients with CAD, and 33 patients with acute coronary syndromes (ACS). Their results presented significantly lower level of EPCs in peripheral blood of patients with CAD compared with control, with no significant difference in patients with ACS, however.

Depression increases the probability of cardiovascular complications, and the quantities of EPCs in peripheral blood of patients with CAD are significantly reduced. The changes in quantities of EPCs in patients with depression may be related to its capability of inducing CAD. In stable angina patients, the percentage of circulating CD34^+^/VEGFR2^+^ EPCs and artery flow-mediated dilation in Subjects with high depression or stress score were significantly lower than that in subjects with normal depression or stress scores (Chen et al., 2013). Di Stefano et al. (2014) investigated that the number of EPCs in ACS with major depressive episodes showed significant decrease compared with that in ACS without major depressive episodes. And then Felice et al. (2015) found that circulating CD34^+^CD133^+^KDR^+^ EPCs levels in ACS with affective disorders were significantly lower than that in ACS s without the affective disorder.

In the above studies, there was no study on EPCs numbers between depression group and depression subjects with cardiovascular diseases. Whether the number and functions of EPCs predict susceptibility to depression in patients with cardiovascular disease or predicts susceptibility to cardiovascular disease in patients with depression, requires an extensive large sample of clinical research.

We summarized above that EPCs were involved in the development of depression. In addition to depression itself, there are also many pathological conditions such as aging and inflammatory diseases associated with the development of depression. Because of special plasticity of CD34^+^ cells, CD34^+^ cells often indicate a distinct subset of cells with progenitor activity (Sidney et al., 2014). Compared with double- or triple-staining in detecting cardiovascular risk, circulating CD34^+^ cells showed more associated with cardiovascular parameters (Fadini et al., 2006). Mandraffino et al. (2012) observed 100 octogenarians for 7 years, CD34^+^ cells play an important role in predicting mortality in the elderly. Further studies confirm that the lower levels of circulating CD34^+^ cells are correlated with increased all causes of deaths, including cardiovascular deaths (Mandraffino et al., 2017). Thus circulating CD34^+^ cells may be as a marker of health. A total of 4,493 participants without cardiovascular disease were followed for 6 years for the development of CHD and mortality, and results showed that depressive symptoms could be an independent risk factor for CHD development and total mortality (Ariyo et al., 2000). Whether circulating CD34^+^ cells are associated with depression would be further investigated. A prospective cohort of elderly participants would be needed to be enrolled in mental health study to testify that circulating CD34^+^ cells may be a marker of mental health and longevity.

**FIGURE 2:**
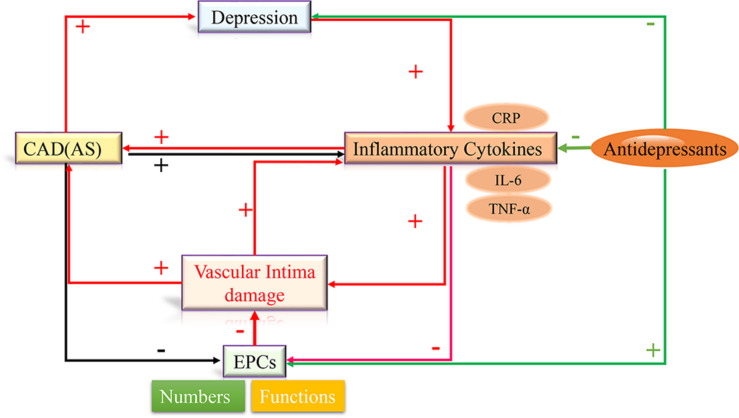
Inflammatory cytokines, depression, CAD, and EPCs dysfunction generate a positive feedback loop. Depression increases the levels of circulating inflammatory cytokines, such as CRP, TNF-α, IL-6 etc., which damage vascular intima, decrease the number of EPCs, and inhibit the functions of EPCs. Dysfunction of EPCs cannot repair the impaired vascular intima, which further stimulates the release of inflammatory factors and induces the development of atherosclerotic cardiovascular. Antidepressants, that inhibit inflammatory cytokines, may reduce the risk of mortality from CAD through improving EPCs functions and repairing intima damage.

There are close correlations among inflammatory factors, depression, CAD, and EPCs, Labib et al. (2019) speculated that pro-inflammatory cytokines-induced dysfunction of circulating EPCs could establish links between depression and atherosclerotic cardiovascular disease. Based on the above conclusions, we propose the hypothesis that depression increases the levels of circulating inflammatory cytokines, such as CRP, TNF-α, IL-6 etc., which damage vascular intima, decrease the number of EPCs, and inhibit the function of EPCs. Dysfunction of EPCs cannot repair the impaired vascular intima, which further stimulates the release of inflammatory factors and induces the development of atherosclerotic cardiovascular and cerebrovascular diseases. Consequently, the development of atherosclerosis and persistent inflammation further aggravate EPCs dysfunction and depression progress. Therefore, inflammatory factors, depression, CAD, and EPCs dysfunction generate a positive feedback loop. Improvement of EPCs numbers and functions may repair the impaired vascular intima, and inhibited the progress of the above positive feedback loop. Antidepressants, that inhibit inflammatory cytokines, may reduce the risk of mortality from CAD through improving EPCs functions and repairing intima damage (see Figure 2).

## Conclusion

Through studying the relationship between EPCs and depression, it was found that the quantity of EPCs was negatively correlated with the severity of depression. And their quantity can be used as an important indicator to predict the occurrence and development of CAD (Ariyo et al., 2000; Werner et al., 2005; Schmidt-Lucke et al., 2019). In the future, EPCs may also serve as an indicator to predict the severity of CAD in depression patients and target for depression patients with CAD. In addition, whether depression affects the quantity and function of EPCs through some specific inflammatory mediators or other diseases such as cardiovascular disease, and then affects the physiology and psychology of human body needs to be further clarified. These unknown factors may become an important research direction for depression targeted prediction and treatment.

## Author Contributions

YT and GL designed and revised the contents of the manuscript. NY and SS wrote the manuscript and answered the revision. GD and KL designed the figures and searched for references. CL, LZ, and JY made the table and searched for references. All authors listed have made a substantial, direct and intellectual contribution to the work, and approved it for publication.

## Conflict of Interest

The authors declare that the research was conducted in the absence of any commercial or financial relationships that could be construed as a potential conflict of interest.

## Publisher’s Note

All claims expressed in this article are solely those of the authors and do not necessarily represent those of their affiliated organizations, or those of the publisher, the editors and the reviewers. Any product that may be evaluated in this article, or claim that may be made by its manufacturer, is not guaranteed or endorsed by the publisher.
